# Nutritional Modulation in Aging and Metabolic Syndrome: Combating Obesity, Vascular Disease, and Frailty

**DOI:** 10.3390/nu18172799

**Published:** 2026-08-27

**Authors:** Sérgio Henrique Sousa Santos, Carla Jeane Aguiar

**Affiliations:** 1 Laboratory of Health Science, Postgraduate Program in Health Sciences, Universidade Estadual de Montes Claros (Unimontes), Montes Claros 39401-089, Brazil; 2 Institute of Agricultural Sciences (ICA), Postgraduate Program in Food and Health, Universidade Federal de Minas Gerais (UFMG), Montes Claros 39404-547, Brazil

Metabolic syndrome (MetS) represents one of the major public health challenges of our time [1]. The coexistence of central obesity, insulin resistance, hypertension, dyslipidemia, and chronic low-grade inflammation markedly increases the risk of cardiovascular disease, type 2 diabetes, functional decline, and premature mortality [2]. As life expectancy continues to increase, the metabolic changes are becoming increasingly intertwined with biological processes associated with aging, such as mitochondrial dysfunction and the reprogramming of cell metabolism, highlighting the importance of effective preventive and therapeutic strategies that simultaneously address metabolic dysfunction and age-related vulnerability [3].

Nutrition has emerged as a central component of this effort. Beyond its traditional role in maintaining energy balance, nutrition influences multiple biological systems involved in metabolic health, including the gut microbiota, immune function, oxidative stress, vascular homeostasis, and cellular metabolism [4,5]. Advances in nutritional science have also strengthened interest in functional foods, bioactive compounds, probiotics, and lifestyle interventions as complementary approaches for promoting healthy aging and reducing cardiometabolic risk [6].

This Special Issue, “**Nutritional Modulation in Aging and Metabolic Syndrome: Combating Obesity, Vascular Disease and Frailty**”, brings together six original contributions that collectively illustrate the breadth of the current research in this field. Although each study addresses a distinct aspect of metabolic health, they converge on a common objective: identifying nutritional and lifestyle strategies that modify the biological pathways associated with obesity, vascular dysfunction, frailty, and aging.

In a pilot clinical trial, Ermolenko et al. (contribution 1) explored the therapeutic potential of autoprobiotic supplementation in individuals with MetS. Using indigenous *Enterococcus* strains demonstrated favorable effects on anthropometric parameters, glycemic control, lipid metabolism, and gut microbiome composition. These findings contribute to the growing body of evidence suggesting that microbiota-directed interventions represent promising strategies for improving metabolic homeostasis [7,8,9].

Vascular aging, a hallmark of cardiometabolic disease, was the focus of two complementary longitudinal studies by Alonso-Diaz et al. (contributions 2 and 3). In the first, higher dietary fiber intake was associated with slower progression of arterial stiffness, whereas increased alcohol and iron consumption correlated with less favorable vascular outcomes over five years. The second study extends these observations by demonstrating inverse associations between the dietary intake of folate (vitamin B9) and vitamin C and the progression of central and peripheral arterial stiffness. Together, these investigations reinforce the concept that specific dietary components contribute to preserving vascular function throughout aging.

The clinical management of obesity was addressed in a randomized, double-blind, placebo-controlled trial conducted by Barbosa et al. (contribution 4). They evaluated the effects of gallic acid supplementation combined with structured physical exercise, which produced improvements in abdominal adiposity, body composition, and selected biochemical markers. The results highlight the potential benefits of integrating naturally occurring polyphenols with exercise-based interventions to optimize metabolic health.

The importance of biological timing in metabolic regulation was examined by Wang et al. (contribution 5). They investigated the interaction between exercise and circadian rhythms in an experimental model of obesity. Their findings reveal distinct tissue-specific adaptations according to the timing of physical activity, suggesting that the metabolic response to exercise is influenced by not only its intensity and duration but also the circadian phase during which the activity is performed. These observations provide new perspectives for the development of chronobiology-informed therapeutic strategies.

This Special Issue concludes with a population-based investigation by Kunutsor et al. (contribution 6), who evaluated composite biomarkers that integrate metabolic malnutrition and systemic inflammation. The authors found that these multimarker indices independently predict all-cause mortality across multimorbidity levels, emphasizing the clinical value of comprehensive metabolic risk assessment in aging populations and supporting the development of more personalized preventive approaches.

Together, the studies presented in this collection underscore the multidimensional nature of metabolic syndrome and healthy aging. They illustrate how nutrition intersects with vascular biology, the gut microbiota, exercise physiology, circadian regulation, and systemic inflammation, reinforcing the need for integrated and interdisciplinary approaches to prevent chronic disease and preserve functional capacity throughout life. By combining mechanistic insights with clinical and epidemiological evidence, these contributions advance our understanding of nutritional modulation as a key component of precision health and healthy aging.

As Guest Editors, we sincerely thank all authors for their valuable contributions and the reviewers for their thoughtful evaluations and commitment to scientific excellence. We are equally grateful to the editorial team of *Nutrients* for their continuous support throughout the preparation of this Special Issue. We hope that the studies presented here will stimulate further research and foster new collaborative efforts aimed at translating nutritional science into effective strategies for improving metabolic health and promoting healthy aging.

## References

[1] Dhondge R.H., Agrawal S., Patil R., Kadu A., Kothari M. (2024). A Comprehensive Review of Metabolic Syndrome and Its Role in Cardiovascular Disease and Type 2 Diabetes Mellitus: Mechanisms, Risk Factors, and Management. Cureus.

[2] Gusev E., Sarapultsev A., Zhuravleva Y. (2026). Insulin Resistance and Inflammation. Int. J. Mol. Sci..

[3] Wei P., Zhang X., Yan C., Sun S., Chen Z., Lin F. (2025). Mitochondrial Dysfunction and Aging: Multidimensional Mechanisms and Therapeutic Strategies. Biogerontology.

[4] Guimarães V.H.D., Marinho B.M., Motta-Santos D., Mendes G.R.L., Santos S.H.S. (2023). Nutritional Implications in the Mechanistic Link between the Intestinal Microbiome, Renin-Angiotensin System, and the Development of Obesity and Metabolic Syndrome. J. Nutr. Biochem..

[5] Aslam H., Trakman G., Dissanayake T., Todd E., Harrison P., Alby C., Jabeen T., Gamage E., Travica N., Marshall S. (2026). Dietary Interventions and the Gut Microbiota: A Systematic Literature Review of 80 Controlled Clinical Trials. J. Transl. Med..

[6] Beaver L.M., Jamieson P.E., Wong C.P., Hosseinikia M., Stevens J.F., Ho E. (2025). Promotion of Healthy Aging Through the Nexus of Gut Microbiota and Dietary Phytochemicals. Adv. Nutr..

[7] Mederle A.L., Dima M., Stoicescu E.R., Căpăstraru B.F., Levai C.M., Hațegan O.A., Maghiari A.L. (2024). Impact of Gut Microbiome Interventions on Glucose and Lipid Metabolism in Metabolic Diseases: A Systematic Review and Meta-Analysis. Life.

[8] Chen K., Wang X., Cao Y., Sun Y., Li X., Liu Y., Zhang Z., Wang Q. (2024). Targeting Gut Microbiota as a Therapeutic Target in T2DM: A Review of Multi-Target Interactions of Probiotics, Prebiotics, Postbiotics, and Synbiotics with the Intestinal Barrier. Pharmacol. Res..

[9] Zhang Z., Mocanu V., Deehan E.C., Hotte N., Zhu Y., Wei S., Kao D.H., Karmali S., Birch D.W., Walter J. (2024). Recipient Microbiome-Related Features Predicting Metabolic Improvement Following Fecal Microbiota Transplantation in Adults with Severe Obesity and Metabolic Syndrome: A Secondary Analysis of a Phase 2 Clinical Trial. Gut Microbes.

